# Cerebral malaria: gamma-interferon redux

**DOI:** 10.3389/fcimb.2014.00113

**Published:** 2014-08-15

**Authors:** Nicholas H. Hunt, Helen J. Ball, Anna M. Hansen, Loke T. Khaw, Jintao Guo, Supun Bakmiwewa, Andrew J. Mitchell, Valéry Combes, Georges E. R. Grau

**Affiliations:** ^1^Molecular Immunopathology Unit, School of Medical Sciences and Bosch Institute, University of SydneySydney, NSW, Australia; ^2^Vascular Immunology Unit, School of Medical Sciences and Bosch Institute, University of SydneySydney, NSW, Australia

**Keywords:** interferon-gamma, cerebral malaria, platelets, microparticles, immunopathology, CD8^+^T lymphocyte, blood-brain barrier, kynurenine pathway

## Abstract

There are two theories that seek to explain the pathogenesis of cerebral malaria, the mechanical obstruction hypothesis and the immunopathology hypothesis. Evidence consistent with both ideas has accumulated from studies of the human disease and experimental models. Thus, some combination of these concepts seems necessary to explain the very complex pattern of changes seen in cerebral malaria. The interactions between malaria parasites, erythrocytes, the cerebral microvascular endothelium, brain parenchymal cells, platelets and microparticles need to be considered. One factor that seems able to knit together much of this complexity is the cytokine interferon-gamma (IFN-γ). In this review we consider findings from the clinical disease, *in vitro* models and the murine counterpart of human cerebral malaria in order to evaluate the roles played by IFN-γ in the pathogenesis of this often fatal and debilitating condition.

## Scope

It is 25 years since the first demonstration that the pro-inflammatory cytokine interferon-γ (IFN-γ) drives the pathogenesis of experimental cerebral malaria (Grau et al., 1989). It therefore seems appropriate to revisit this topic and evaluate progress in our understanding of the mechanisms involved, as well as their significance for the pathogenesis of this life-threatening (Molyneux et al., 1989; Newton et al., 2000) and disabling (Molyneux et al., 1989; Kihara et al., 2006; John et al., 2008) condition in human beings.

The production of IFN-γ in humans and mice occurs as part of anti-malarial immunity. This role of the cytokine has been reviewed recently (McCall and Sauerwein, 2010) and will not be discussed here. Although IFN-γ also has been shown to play an essential role in the pathogenesis of some other infectious diseases that adversely affect the central nervous system (CNS), such as pneumococcal meningitis (Mitchell et al., 2012), we here will focus on the cerebral manifestations of severe malaria caused by *Plasmodium falciparum* (*Pf*). Furthermore, we will not deal with the possible roles of the Type I interferons in cerebral malaria (Vigario et al., 2007; Morrell et al., 2011; Ball et al., 2013; Palomo et al., 2013).

## Interferon-γ

The *IFN-γ* gene was cloned in 1982, though knowledge of the existence of IFN-γ-like biological activity dates back a further two decades (Billiau and Matthys, 2009). The cytokine has a molecular weight of 45 kDa and its gene is located on chromosome 12 in humans and 10 in mice. It has an enormous range of actions upon many cell types, in particular those involved in immunity, both innate and adaptive, and inflammation. The IFN-γ-producing cells of particular relevance to malaria include CD4^+^, CD8^+^, and γδT lymphocytes, and Natural Killer (NK) cells.

IFN-γ signaling pathways have been well-characterized. The IFN-γ receptor is composed of two chains and binding of the cytokine leads to recruitment of the tyrosine kinases JAK1 and JAK2. This leads to activation of STAT1, which homodimerizes, enters the nucleus and initiates the transcription of Interferon Response Factors that induce the expression of a wide range of genes (Schroder et al., 2004; Saha et al., 2010). Other signal transduction pathways can be triggered by IFN-γ, and the actions of the cytokine are negatively modulated by suppressor of cytokine signaling proteins (Saha et al., 2010). Recombinant IFN-γ and adenovirus vectors that express IFN-γ cDNA have been trialed clinically, with some success, for a range of diseases including chronic granulomatous disease, hepatitis, tuberculosis, and certain cancers (see Miller et al., 2009 for review). Antibodies that neutralize the cytokine's actions have been used to treat rheumatoid arthritis and multiple sclerosis (Miller et al., 2009).

Malaria immunity involves both the innate and adaptive immune systems (Good et al., 2005; Riley et al., 2006). Pro-inflammatory cytokines, in particular IFN-γ, drive the cell-mediated immune response that controls parasite numbers early in the intraerythrocytic cycle, and antibody seems to be responsible for “mopping up” and preventing recrudescence (Good et al., 2005; Riley et al., 2006; McCall and Sauerwein, 2010).

## Severe malaria

Severe malaria is a set of systemic complications associated with *Pf* infection that includes cerebral malaria, which is involved in a high proportion of fatal cases, particularly in African children. This acute brain dysfunction leads to coma and, in the absence of anti-malarial therapy, death. Fortunately, this occurs in only a small percentage of *Pf* infections. Nevertheless, long-term neurological sequelae occur in a substantial proportion of those who survive pediatric cerebral malaria (Molyneux et al., 1989; Kihara et al., 2006; John et al., 2008).

**Figure 1:**
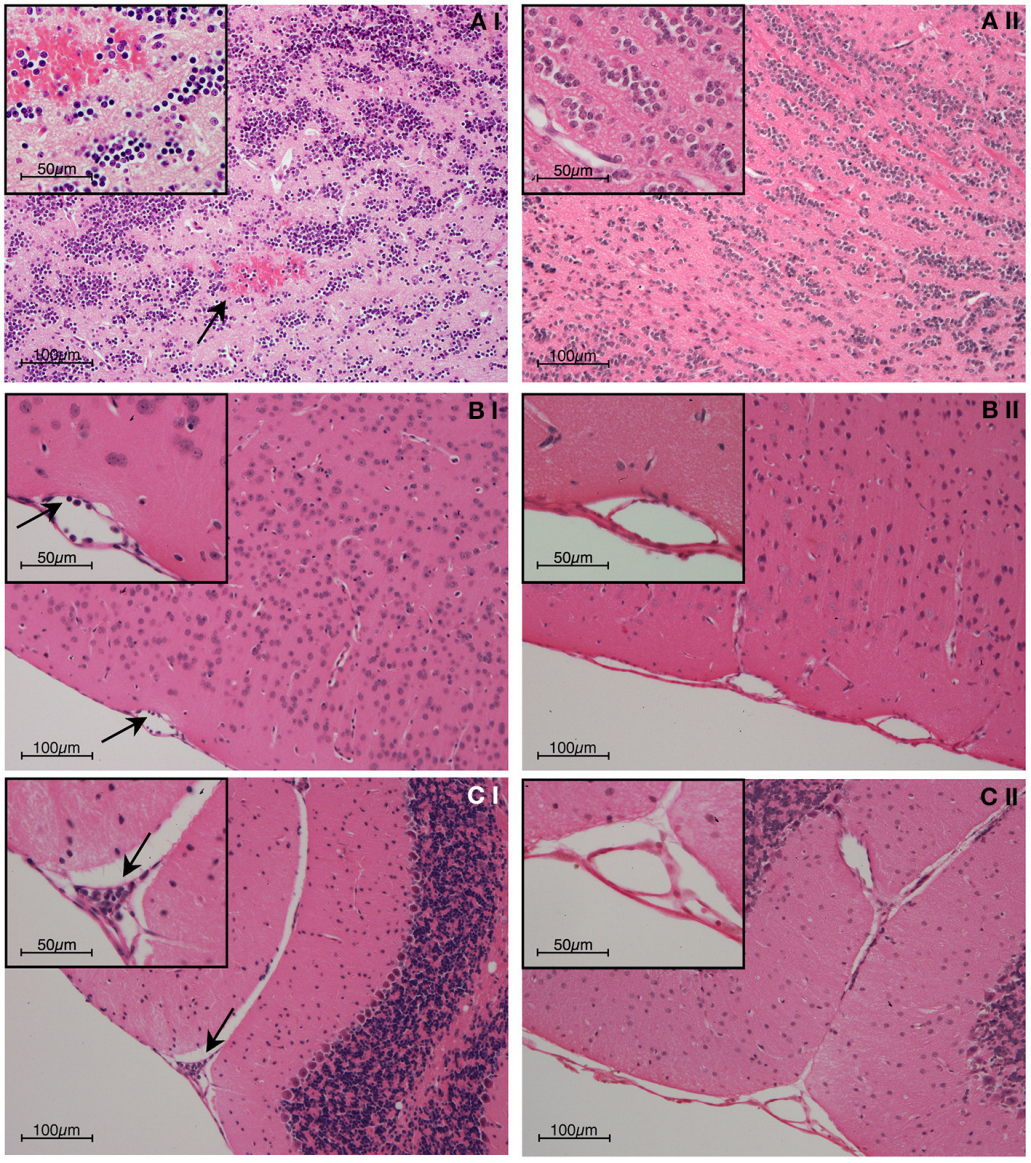
**Representative post-mortem histopathology findings in H & E stained brain sections from (i) wild-type and (ii) IFN-γ^−/−^ C57BL/6 mice on day 6 post-inoculation with 1 × 10^6^ PbA-PRBC**. As no difference was evident between uninfected mice and infected IFN-γ^−/−^ mice, only the latter are shown. **(A)** Olfactory bulb; **(B)** Meningeal vessel; **(C)** Cerebellum. The brains of PbA-infected w/type mice showed hemorrhage and leukocyte adhesion to the cerebral vasculature (arrows), whereas no pathological findings were evident in any IFN-γ^−/−^ mouse. In this and later Figures (where appropriate) the work was carried out according to national and State legislation on animal experimentation, with approval from the University of Sydney Animal Ethics Committee.

**Figure 2:**
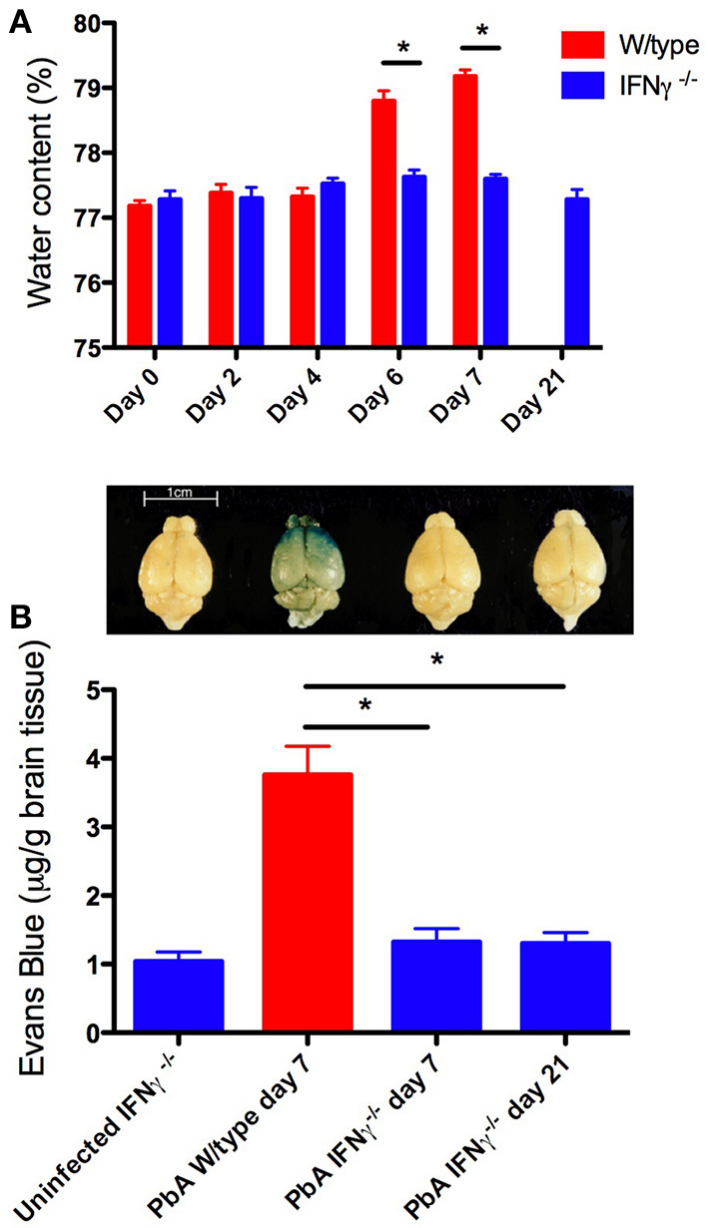
**Brain edema and blood-brain barrier compromise after PbA infection**. Water content was calculated from wet and dry weight. Evans blue, a dye that binds to circulating albumin, was injected intravenously 2 h before mice were euthanased; the brain was perfused with saline, removed, photographed, and water-extracted; the Evans blue content was measured spectrophotometrically at 510 nm. **(A)** PbA-infected wild-type mouse brains had significantly greater water content compared with infected IFN-γ^−/−^ mice at days 6 and 7 post-inoculation (^*^p < 0.001, Two-Way ANOVA with Bonferroni post-test). **(B)** PbA-infected wild-type mice had significantly greater extravasation of Evans Blue dye into the brain parenchyma on day 7 post-inoculation compared to infected IFN-γ^−/−^ mice on day 7 or 21 post-inoculation (^*^p < 0.001, One-Way ANOVA with Bonferroni post-test). Above each bar of the graph is shown a representative brain from that experimental group. Columns and vertical bars are mean ± s.e.m. (*n* = 5 per group).

**Figure 3:**
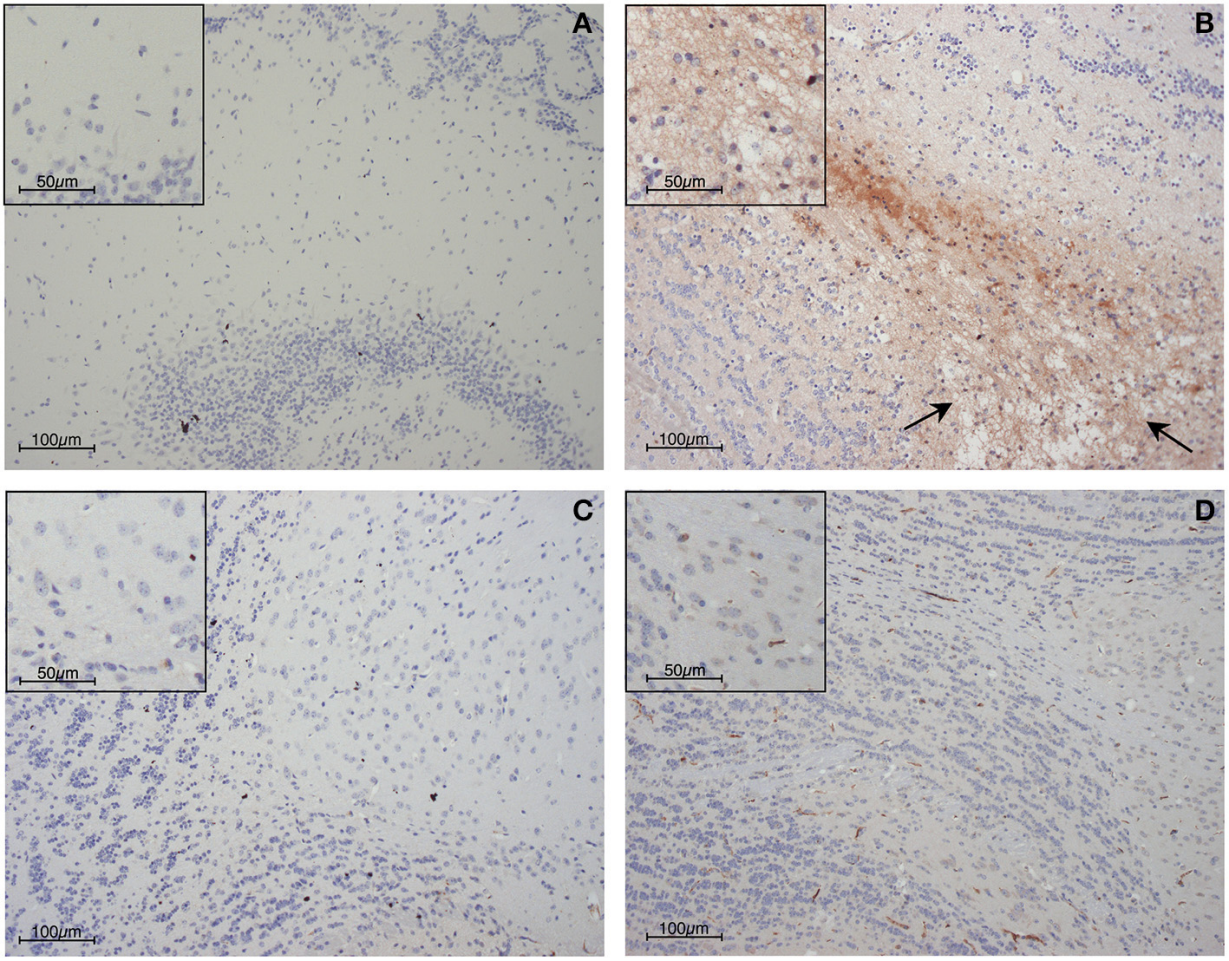
**Blood-brain barrier compromise during PbA infection, as determined by immunohistochemical detection of fibrinogen within the parenchyma of the olfactory bulb. (A)** Uninfected control mouse; **(B)** PbA-infected wild-type mouse at day 6 post-inoculation; **(C)** PbA-infected IFN-γ^−/−^ mouse at day 6 post-inoculation and **(D)** day 20–22 post-inoculation. Blood-brain barrier permeabilization to protein is clearly evident within the wild-type mouse, in which edematous changes also can be seen (arrows). These changes were not seen in IFN-γ^−/−^ mice at any stage of infection.

Histopathological observations and other evidence have established that hemorrhage, sequestration of parasitized red blood cells (PRBC) and leukocytes, and increased blood-brain barrier permeability occur in both human and murine cerebral malaria (Toro and Roman, 1978; Thumwood et al., 1988; Das et al., 1991; Chan-Ling et al., 1992; Patnaik et al., 1994; Turner et al., 1994; Brown et al., 1999a, 2001; White et al., 2001; van der Heyde et al., 2001; Adams et al., 2002; Grau et al., 2003; Hunt and Grau, 2003; Taylor et al., 2004; Amante et al., 2010; Claser et al., 2011; Cunnington et al., 2013). Examples of these phenomena from the experimental model are shown in Figures 1–3.

Two mechanisms are considered to be important in CM pathogenesis: microvascular obstruction leading to hypoxia, and immunopathological processes. Although some proponents of the former mechanism discount the latter (White et al., 2013), many researchers in the field consider that a combination of these two best explains the outcomes of many published clinical, pathological, experimental and genetic investigations of cerebral malaria (Grau and De Kossodo, 1994; Hunt and Grau, 2003; Mackintosh et al., 2004; Cunnington et al., 2013).

Microvascular obstruction in the brain occurs in human cerebral malaria. This is thought to be a consequence of binding of *Pf*-parasitized erythrocytes (*Pf*-PRBC) to the vascular endothelium through a parasite-encoded protein, *Pf*EMP1, that can interact with a number of adhesion molecules (Warrell et al., 1988; Berendt et al., 1994; Turner et al., 1994, 2013). Accumulation of *Pf*-PRBC in brain microvessels is characteristic of pediatric (Taylor et al., 2004) and adult (Ponsford et al., 2012) cerebral malaria victims, with leukocytes also being present (Patnaik et al., 1994; Grau et al., 2003; Taylor et al., 2004; Armah et al., 2005), and is accompanied by lactate accumulation in the cerebrospinal fluid (CSF) (White et al., 1985; Warrell et al., 1988; Molyneux et al., 1989), consistent with the suggestion that oxygen supply to the brain is compromised.

Microvascular obstruction (Chan-Ling et al., 1992), hypoxia (Hempel et al., 2011) and accumulation of lactate in the CNS (Sanni et al., 2001; Rae et al., 2004) also occur in experimental cerebral malaria [*P. berghei* ANKA (PbA) infection in mice]. Reduced blood flow and metabolic changes consistent with hypoxia also have been identified in this mouse model using multimodal magnetic resonance (Penet et al., 2005). The cause of vascular obstruction in murine cerebral malaria has not been established, though leukocytes and PbA-PRBC accumulate in the CNS microcirculation (Thumwood et al., 1988; Chan-Ling et al., 1992; Amante et al., 2007; Miu et al., 2008a; Nie et al., 2009; Ampawong et al., 2014).

Thus, a hypoxic metabolic profile and accumulation of PRBC and leukocytes in the cerebral microcirculation are features of both human and experimental CM. One of the great, unanswered questions is why vascular obstruction does not lead to catastrophic neuronal damage, as it does in stroke. One possibility is that any occlusive events and ischemia are very short-lived, which is difficult to test. Another is that the hypoxic metabolic profile is also influenced by processes independent of vascular obstruction, such as the actions of cytokines (Rae et al., 2004; Parekh et al., 2006).

Administration of an IFN-γ-neutralizing antibody led to the concept that this cytokine is a key contributor to the pathogenesis of cerebral malaria in the PbA model (Grau et al., 1989). This finding subsequently was confirmed with IFN-γ gene knockout (GKO) (Yanez et al., 1996; Sanni et al., 1998; Belnoue et al., 2008) and IFN-γ receptor GKO (Amani et al., 2000) mice. There is substantial evidence implicating other pro-inflammatory cytokines in the pathogenesis of cerebral malaria, both human and experimental (Clark and Rockett, 1994; Udomsangpetch et al., 1997; Brown et al., 1999b; Engwerda et al., 2002; Hunt and Grau, 2003; Schofield and Grau, 2005; Hunt et al., 2006). Human genetic association studies have demonstrated links between immune cell products and susceptibility to human cerebral malaria (Kwiatkowski, 2005; Verra et al., 2009). Anti-inflammatory molecules or processes, such as interleukin (IL)-10 (de Kossodo et al., 1997; Ho et al., 1998a), transforming growth factor-β (Omer and Riley, 1998; Riley et al., 2006) and regulatory T cells (Nie et al., 2007), appear to be important in malaria for “damping down” innate immune responses and channeling the development of effective adaptive immunity.

Interaction between microvascular obstruction and immunopathology might occur in a number of ways. First, pro-inflammatory cytokines, including IFN-γ, induce the expression on endothelial cells of adhesion molecules (Wahl et al., 1996; Weiser et al., 2007), which are capable of mediating *Pf*-RBC and leukocyte interactions with the endothelium (Wahl et al., 1996; Ho et al., 1998b). Indeed, IFN-γ and lymphotoxin α, the two key pathogenetic cytokines in experimental cerebral malaria (Grau et al., 1989; Engwerda et al., 2002), are strongly synergistic in inducing the expression of vascular cell adhesion molecule-1, intercellular adhesion molecule-1 (ICAM-1) and E-selectin in mouse brain endothelial cells *in vitro* (Weiser et al., 2007). Expression of these adhesion molecules on the cerebral microvascular endothelium has been reported in human and murine cerebral malaria (de Kossodo and Grau, 1993; Turner et al., 1994; Bauer et al., 2002; Armah et al., 2005) and ICAM-1 GKO mice are protected against PbA-induced cerebral malaria (Favre et al., 1999).

**Figure 4:**
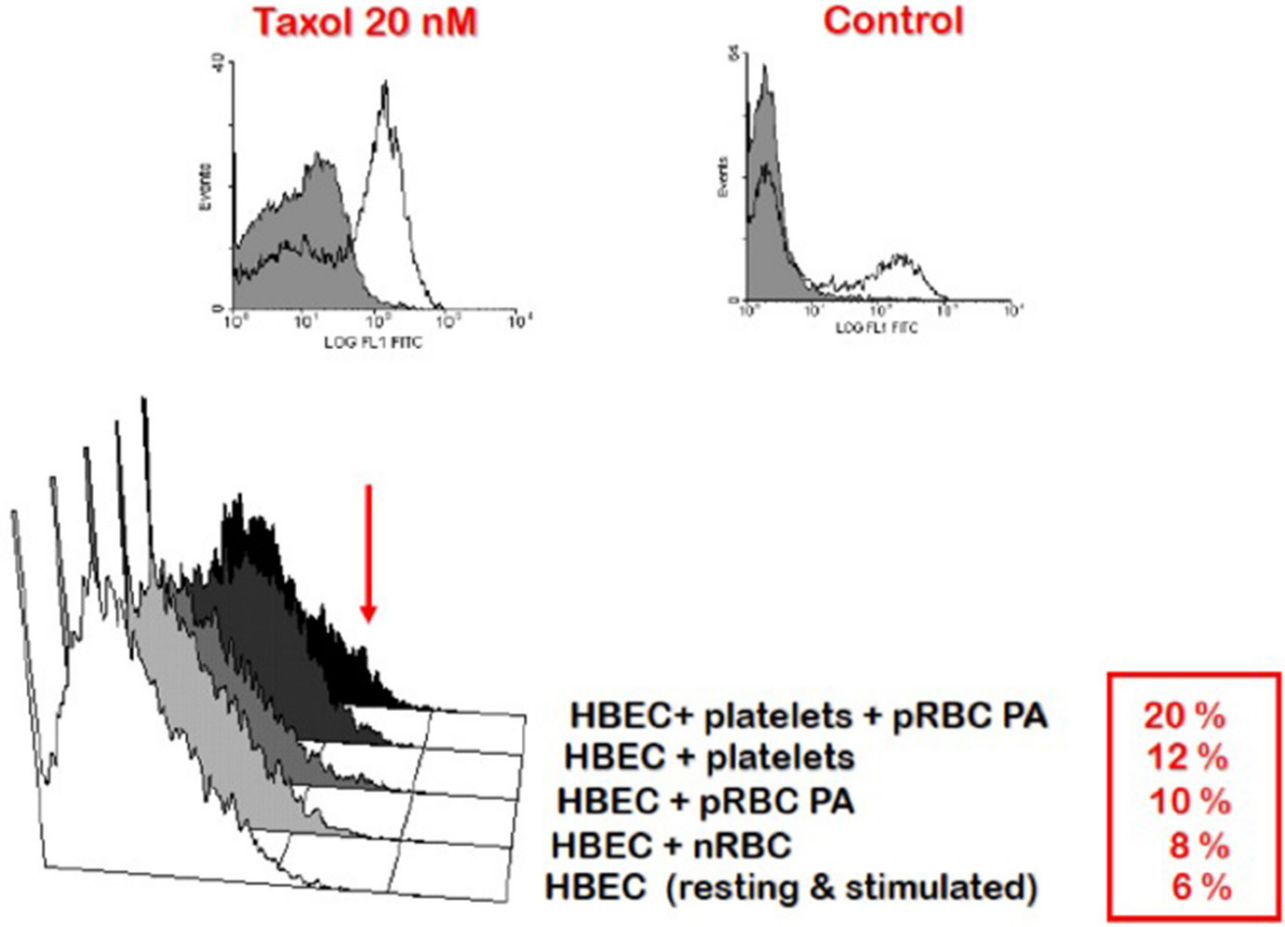
**Enhancement of platelet-mediated endothelial cell apoptosis after IFN-γ stimulation**. HBEC were stimulated with IFN-γ overnight prior to addition of platelets and RBC. Taxol treatment of HBEC was used as the positive control. FITC-BrdU nuclear fragmentation was quantified using the APO-Direct Kit (BD Biosciences) and an EPICS-XL flow cytometer (Beckman-Coulter). Results are expressed as percentages of cells undergoing apoptosis. HBEC, human brain endothelial cells; nRBC, normal red blood cells; pRBC, parasitized red blood cells; PA, Palo Alto strain of *Plasmodium falciparum*. In this and later Figures (where appropriate) the work was carried out according to national and State legislation on human experimentation, with approval from the University of Sydney Human Ethics Committee.

Second, the accumulation of PRBC and leukocytes in the cerebral microcirculation favors their interaction and might serve to focus the production of immune/inflammatory products, such as pro-inflammatory cytokines, in that critical location (Khaw et al., 2013). This setting has been modeled *in vitro* using co-cultures of mouse (El-Assaad et al., 2013) and human (Wassmer et al., 2004, 2006a,b) brain endothelial cells and PRBC. Here, cytokines, including IFN-γ, tumor necrosis factor (TNF) and lymphotoxin α, lead to endothelial cell activation resulting in the local binding of platelets, which, in turn, enhances endothelial activation and apoptosis (Wassmer et al., 2006a,b) (Figure 4), as discussed in more detail below.

Finally, we recently have proposed that the effects of hypoxia and cytokines might intersect at key locations in the pathogenesis of cerebral malaria, namely endothelial cells and astrocytes (Combes et al., 2012), which are key components of the neurovascular unit. Astrocytes influence the functions of all CNS cells and have unique features that make them a strong candidate to be a convergence point of ischemia and immunopathology in the events leading to cerebral malaria (Combes et al., 2012). They control the extracellular milieu of the CNS, modulate synaptic transmission, act as a bioenergetic regulator and influence vascular properties, including blood-brain barrier integrity and blood flow. Their central roles in these vital functions mean that astrocytes often are a major determinant of the outcome of several diseases that affect the CNS (Verkhrasky et al., 2009). For example, in stroke, obstruction of an artery prevents oxygen and glucose delivery to the downstream tissue. In the core of the ischemic region, where the supply deficit is most severe, astrocytes and neurons perish through a network of interrelated processes, many of which are due to severe loss of intracellular ATP (Rossi et al., 2007). Surrounding this core is the hypoperfused penumbral region, in which cellular ATP is less compromised. It is now believed that astrocytes hold the key to whether neurons die, or regain function, in the penumbral region in stroke (Nedergaard and Dirnagl, 2005; Panickar and Norenberg, 2005; Trendelenburg and Dirnagl, 2005; Takano et al., 2009). It is possible that they have similar significance in cerebral malaria, where the occurrence of neuronal damage has been reported (Medana et al., 2002, 2007).

Astrocytes are target cells of IFN-γ, with outcomes such as astrogliosis and production of chemokines (John et al., 2003; Liberto et al., 2004). Changes in astrocyte morphology and function occur early in the course of experimental cerebral malaria (Medana et al., 1996), as visualized in retinal wholemounts. The retina parallels the pathological changes in the brain in both human and experimental cerebral malaria (Chan-Ling et al., 1992; White et al., 2009), and retinal changes have considerable diagnostic and pathophysiological significance in the human condition (Beare et al., 2004; White et al., 2009; Birbeck et al., 2010). Astrogliosis in murine malaria is seen in cerebral malaria but not in severe anemia (Medana et al., 1996; Ampawong et al., 2014). Accompanying this astrocyte activation is production of CXCL10 (Miu et al., 2008a). Morphological changes in astrocytes have been reported in the brain and retina in human severe malaria (Medana et al., 2002; White et al., 2009), which, by analogy with other CNS diseases (Panickar and Norenberg, 2005; Rossi and Volterra, 2009), could be in part a response to the occurrence of neuronal damage. Local production of cytokines and a hypoxic environment also might play a role, as discussed above.

**Figure 5:**
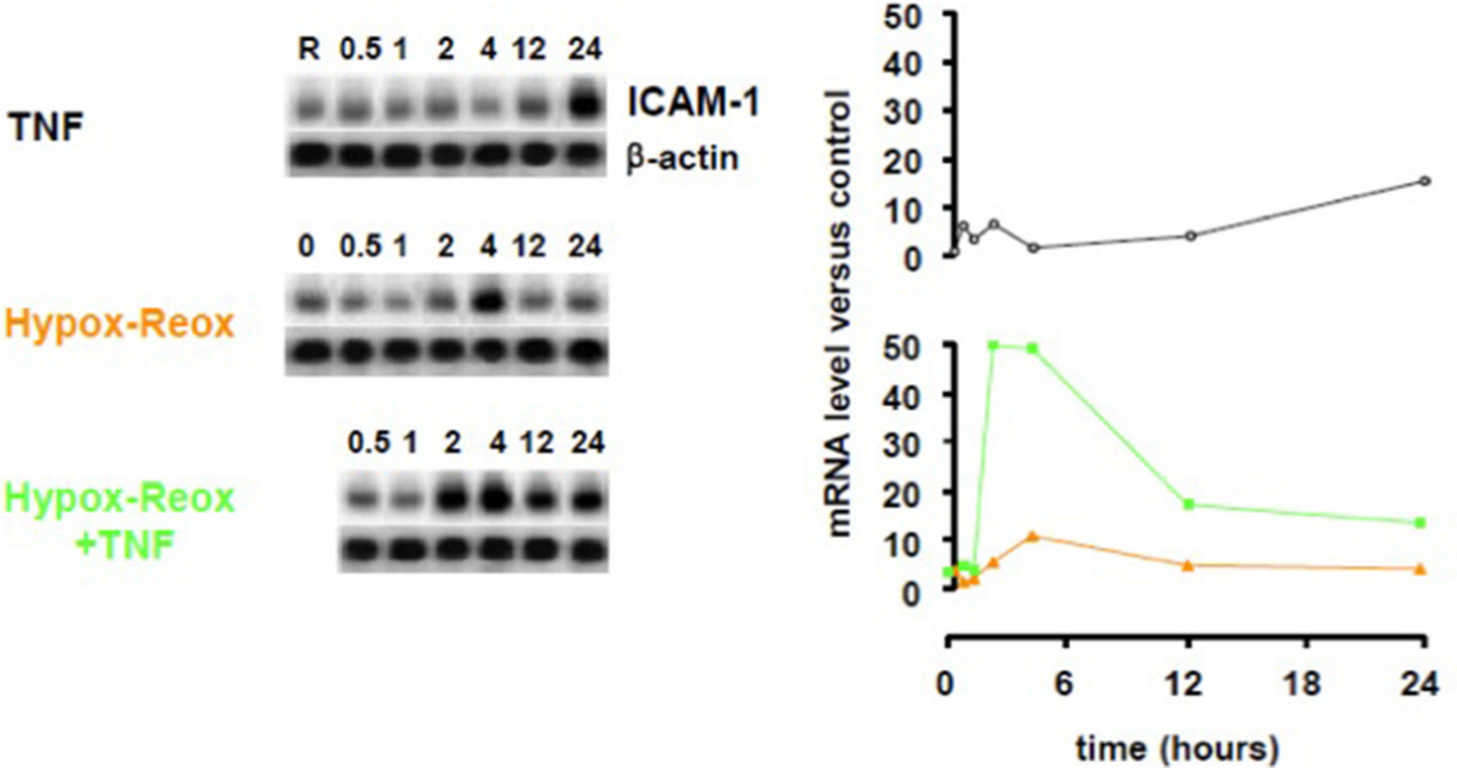
**Effect of hypoxia-reoxygenation on TNF-induced ICAM-1 upregulation in human brain microvascular endothelial cells**. Human brain endothelial cells (HBEC) were exposed to 1% O_2_ for 18 h, then returned to normoxia, stimulated or not with 50 ng/mL TNF and ICAM-1 mRNA was quantified at the designated time points using a PhosphorImager® SI (Molecular Dynamics). TNF, tumor necrosis factor; ICAM-1, intercellular cell adhesion molecule-1.

Endothelial cells are another pivotal cell type that affects the CNS in infectious diseases (Combes et al., 2012). The endothelium becomes activated in cerebral malaria and its roles include the expression of adhesion molecules (de Kossodo and Grau, 1993; Turner et al., 1994; Favre et al., 1999; Bauer et al., 2002; Armah et al., 2005), production of chemokines (Miu et al., 2008a), release of microparticles (Combes et al., 2006, 2010), generation of pro-coagulant factors (Grau et al., 1997) and interactions with platelets (Lou et al., 1997; Wassmer et al., 2006a) (Figure 4). Several of these processes are stimulated by IFN-γ, and endothelial dysfunction is induced by hypoxia in other conditions (Jelic and Le Jemtel, 2008). Hypoxia/reoxygenation was found to dramatically enhance the stimulatory effect of TNF on ICAM-1 upregulation in human brain microvascular endothelial cells (Figure 5).

Thus, the activities of astrocytes and endothelial cells are influenced by both pro-inflammatory cytokines and hypoxia, processes that are widely considered to be relevant in the context of cerebral malaria.

## Source of IFN-γ in severe malaria

In human volunteers infected with *Pf* sporozoites, IFN-γ is initially detectable at around one to two days after initiation of blood stage infection (Walther et al., 2006). These processes have been modeled *in vitro* using co-culture of *Pf*-PRBC and human peripheral blood mononuclear cells (HPBM) from malaria-naïve donors. In this system, IFN-γ is produced with similar kinetics to that seen *in vivo* (Artavanis-Tsakonas and Riley, 2002). Some studies have argued that γδT cells expressing NK cell receptors may be the dominant source of IFN-γ (Hensmann and Kwiatkowski, 2001; D'Ombrain et al., 2007). However, substantial evidence supports a model in which the parasite is initially sensed by myeloid cells, which in turn stimulate NK cells to produce IFN-γ via contact and cytokine signals (Artavanis-Tsakonas and Riley, 2002; Baratin et al., 2005; Korbel et al., 2005; Newman et al., 2006). Following this initial production of IFN-γ by NK cells (within the first 24 h in culture), αβT cells may then dominate the IFN-γ response (Horowitz et al., 2010). *In vivo*, some leukocyte-*Pf*-iRBC interactions may take place in the cerebral microcirculation and, interestingly, IFN-γ expression by HPBM is substantially enhanced when co-cultured with *Pf*-PRBC in the presence of brain endothelial cells (Khaw et al., 2013), a process that requires the presence of NK cells.

In experimental murine cerebral malaria the relative contribution of different cell subsets to levels of circulating IFN-γ is largely dependent upon on the stage of infection. Serum levels of IFN-γ at times prior to development of the cerebral complications are at least partially under the control of genes in the Natural Killer Complex loci, which suggests that early IFN-γ derives from either NK or NKT cells (Hansen et al., 2003, 2005, 2014). This early production of IFN-γ by NK cells is dependent upon IL-12 from dendritic cells (Ryg-Cornejo et al., 2013). Use of IFNγ reporter mice reinforced that such production was largely from NK cells, whereas at late stages of infection, immediately prior to and during neurological disease, CD4^+^ and CD8^+^T cells are the predominant sources (Villegas-Mendez et al., 2012).

## Targets and consequences of IFN-γ in cerebral malaria

**Figure 6:**
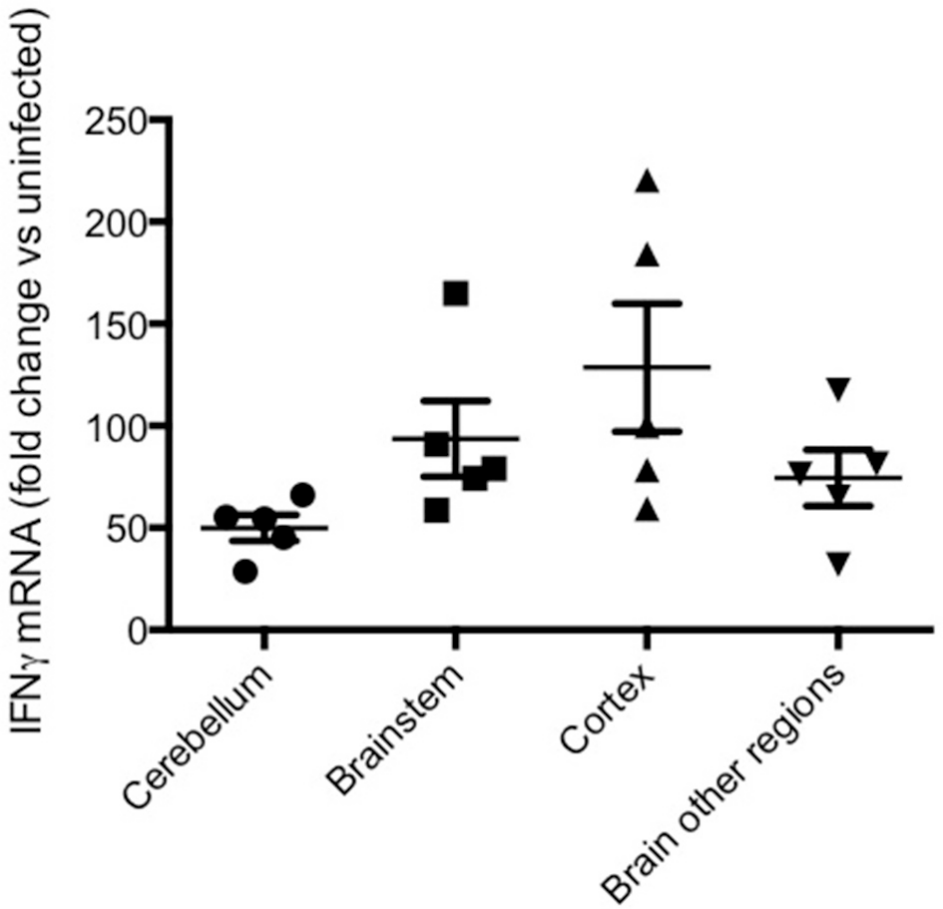
**IFN-γ mRNA in various brain regions in murine cerebral malaria**. C57BL/6 mice were inoculated with 2 × 10^5^ PbA-PRBC, their brains removed on day 6 post-inoculation and dissected into regions prior to homogenization. RT-PCR was performed as described elsewhere (50). Horizontal lines and vertical bars are mean ± s.e.m. of fold differences vs. equivalent samples from uninfected mice.

**Figure 7:**
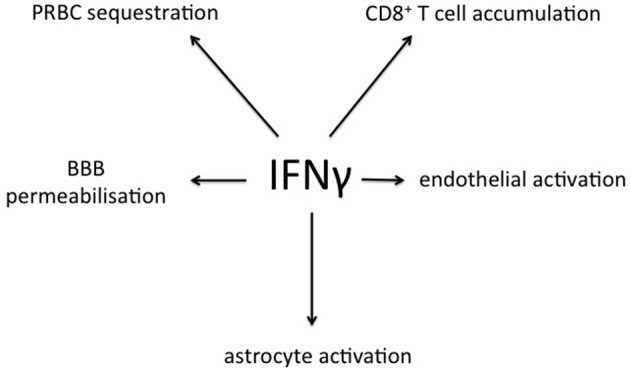
**Summary of processes relevant to cerebral malaria that are stimulated by IFN-γ, as derived from experimental models**. PRBC, parasitized red blood cell; BBB, blood-brain barrier.

In addition to systemic production of IFN-γ, in the experimental model IFN-γ mRNA is strongly expressed in brain homogenates late in the course of PbA infection, with differences of degree between various brain regions (Figure 6). The IFN-γ receptor is widely expressed throughout the hemopoetic, cardiovascular and CNSs, providing many targets for this cytokine in cerebral malaria (Figure 7). Many cell populations that have been implicated in the pathogenesis of the condition may respond to IFN-γ, for example various types of leukocytes, endothelial cells and brain parenchymal cells such as astrocytes and microglia. These have been extensively studied in model systems, both *in vivo* and *in vitro*. For obvious reasons this type of intervention-driven hypothesis testing is impossible in human cerebral malaria, which sometimes has led to skepticism about the roles of IFN-γ and other cytokines in the human disease. Of course, this caveat applies equally to every other hypothesis about the pathogenesis of severe malaria. Gene expression analysis in experimental cerebral malaria has revealed the induction of many IFN-γ-dependent genes in the CNS (Lovegrove et al., 2007; Miu et al., 2008b) and an equivalent post-mortem study in the human condition is a worthwhile goal.

During PbA infection, current evidence supports a model in which recruitment of leukocytes, in particular CD8^+^T cells, by IFN-γ-dependent processes is a key outcome. Blood-brain barrier compromise in experimental cerebral malaria clearly is driven by IFN-γ since it is greatly attenuated in GKO animals (Figures 1–3). Importantly, coincident accumulation of PbA-PRBC and CD8^+^T cells is essential for pathogenesis (McQuillan et al., 2011). IFN-γ drives sequestration of both PbA-PRBC (Amante et al., 2010; Claser et al., 2011) and CD8^+^T cells (Belnoue et al., 2008; Miu et al., 2008a) in the brain microvasculature. Although leukocytes are commonly found within cerebral microvessels in human and experimental cerebral malaria, there is little entry into the brain parenchyma. However, this is still a form of inflammation, albeit intravascular rather than intratissular. Signals originating within the parenchyma that impact upon immune and inflammatory cells include CXCL10 production by astrocytes (Miu et al., 2008a), as discussed below.

Both CD4^+^ and CD8^+^T lymphocytes play obligatory roles in experimental cerebral malaria (Grau et al., 1986; Yanez et al., 1996; Belnoue et al., 2002; Villegas-Mendez et al., 2012). Parasite antigen-specific CD8^+^ cytotoxic lymphocytes are generated in murine cerebral malaria (Lau et al., 2011), possibly through interactions with Clec9A dendritic cells (deWalick et al., 2007; Lundie et al., 2008; Piva et al., 2012). CD8^+^T cells recruited to the brain microvasculature in this system do not carry out their pathogenetic function through IFN-γ production (Villegas-Mendez et al., 2012). Instead, there is good evidence supporting cross-presentation of malaria antigens on CNS microvascular endothelial cells (Howland et al., 2013) and recognition by CD8^+^ cytotoxic lymphocytes leading to endothelial damage in a perforin- and granzyme B-dependant manner (Potter et al., 1999, 2006; Nitcheu et al., 2003; Haque et al., 2011). This may be the basis of the compromised blood-brain barrier described earlier.

IFN-γ is essential for accumulation of CD8^+^T cells within the brain microvessels during experimental cerebral malaria (Belnoue et al., 2008). IFN-γ produced prior to end stage disease drives production of the CXCR3-binding chemokines CXCL9 and CXCL10 (Campanella et al., 2008; Miu et al., 2008a). There is strong evidence that CXCL9 or CXCL10 and their receptor CXCR3 are required for the development of murine cerebral malaria (Belnoue et al., 2008; Campanella et al., 2008; Van den Steen et al., 2008; Miu et al., 2008a; Nie et al., 2009). NK cells localize to the brain vasculature from around day 4 post-infection and can mediate sequestration of αβT cells in an IFN-γ- and CXCR3-dependent manner (Hansen et al., 2007). Alternatively, adoptive transfer studies using IFNγ-deficient recipients have suggested that IFN-γ produced by CD4^+^T cells is the dominant source of IFN-γ that is involved in induction of CXCR3 ligands, CD8^+^T cell sequestration and development of clinical disease (Villegas-Mendez et al., 2012). Importantly, higher plasma and CSF levels of CXCL10 are seen in Ghanaian children with cerebral malaria, compared to those with severe malaria and non-malaria cases (Armah et al., 2007). Furthermore, polymorphisms in the human *CXCL10* gene that affect plasma CXCL10 correlate with the incidence of cerebral malaria, particularly in males, in a manner consistent with the data from the experimental studies in mice (Wilson et al., 2013). Thus, this IFN-γ-regulated chemokine appears to be involved in the disease process in both human and murine cerebral malaria.

The effects of IFN-γ have been studied in endothelial cell-platelet-PRBC co-cultures, which involved the human brain microvascular endothelial cell line 5i, human platelets from normal donors and the Palo-Alto (PA) strain of *Pf* (Wassmer et al., 2006a). While TNF is only able to upregulate ICAM-1, VCAM-1, and CD40 on endothelial cells, IFN-γ also induces CD36 upregulation (Petzelbauer et al., 1993). As shown in Figure 4, IFN-γ can enhance platelet binding to endothelial surfaces, presumably via CD36 and/or ICAM-1, and increase the proportion of brain endothelial cells that undergo apoptosis, as measured by FITC-BrdU nuclear fragmentation, when compared to those co-cultured with either PRBC or platelets alone. The effect of IFN-γ (20.0% of apoptotic cells) is however weaker than that of lymphotoxin α and TNF (25.5 and 45.2%, respectively) (Wassmer et al., 2006a). Stimulation of brain endothelial cells by IFN-γ also enhances their microparticle release, with different kinetics and response frequencies of cells compared to TNF stimulation (Latham et al., 2013).

Taken together, these data indicate that IFN-γ participates in cerebral malaria pathogenesis by affecting endothelial integrity.

A less commonly known product of the endothelium in cerebral malaria is indoleamine dioxygenase-1 (IDO-1), one of three intracellular enzymes that convert tryptophan into N-formylkynurenine. This is the first step in the kynurenine pathway, which leads to the production of numerous biologically-active molecules (Ball et al., 2009). IDO-1 expression is regulated by IFN-γ. As reviewed previously (Hunt et al., 2006; Combes et al., 2012), the kynurenine pathway is activated in human (Medana et al., 2003) and experimental (Sanni et al., 1998) cerebral malaria. IDO-1 expression is induced by IFN-γ selectively in endothelial cells in murine malaria infections (Hansen et al., 2004). This probably is a tissue protective response, but one that can become dysregulated in the brain during PbA infection, contributing to abnormalities in neuronal function (Hunt et al., 2006). A striking imbalance in kynurenine pathway metabolites in favor of the neuroexcitotoxin quinolinic acid is observed in the mouse brain as the neuronal symptoms develop (Sanni et al., 1998). However, IDO-1 GKO mice are not protected against fatal cerebral malaria (Miu et al., 2009), although pharmacological inhibition of the production of deleterious metabolites through the kynurenine pathway does reduce mortality (Clark et al., 2005; Miu et al., 2009). These somewhat conflicting findings require explanation.

**Figure 8:**
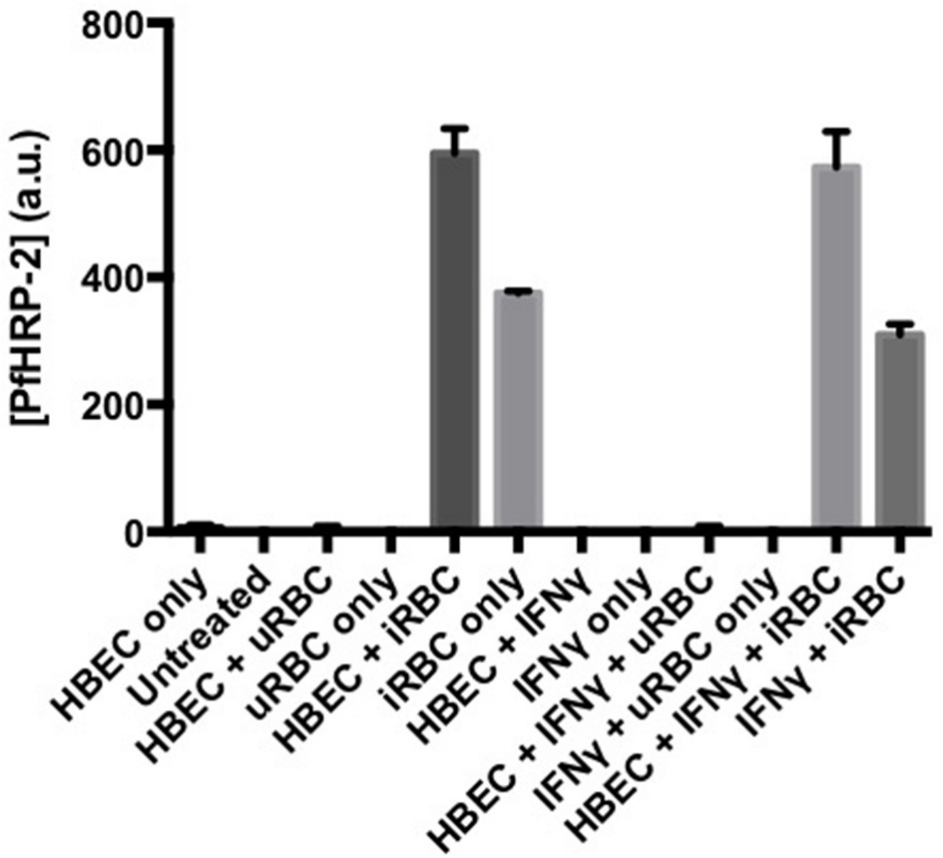
**Activation of endothelial indoleamine dioxygenase-1 by IFN-γ does not affect growth of co-cultured *Plasmodium falciparum***. *Pf*-PRBC or uninfected RBC (uRBC) were cultured together with human brain endothelial cells (line HBEC-5i) for 72 h. Parasite growth as *Plasmodium falciparum* histidine rich protein-2 (*Pf*HRP-2) was determined by ELISA. Under the same conditions, the IFN-γ treatment previously had been demonstrated to deplete tryptophan and cause kynurenine formation (data not shown), indicating expression and activity of IDO-1. Values are mean ± s.e.m. of triplicate determinations in a single experiment.

Activation of the kynurenine pathway in endothelial cells restricts the growth of some bacteria, viruses and parasites (Adam et al., 2005), probably via depletion of tryptophan. However, this is not true of *Pf* (Figure 8). At the systemic level, the IFN-γ/IDO-1/kynurenine axis appears to be an important mechanism contributing to the hypotension associated with murine malaria (Wang et al., 2010), but this has not been investigated to date in the clinical disease.

## Summary and conclusions

**Figure 9:**
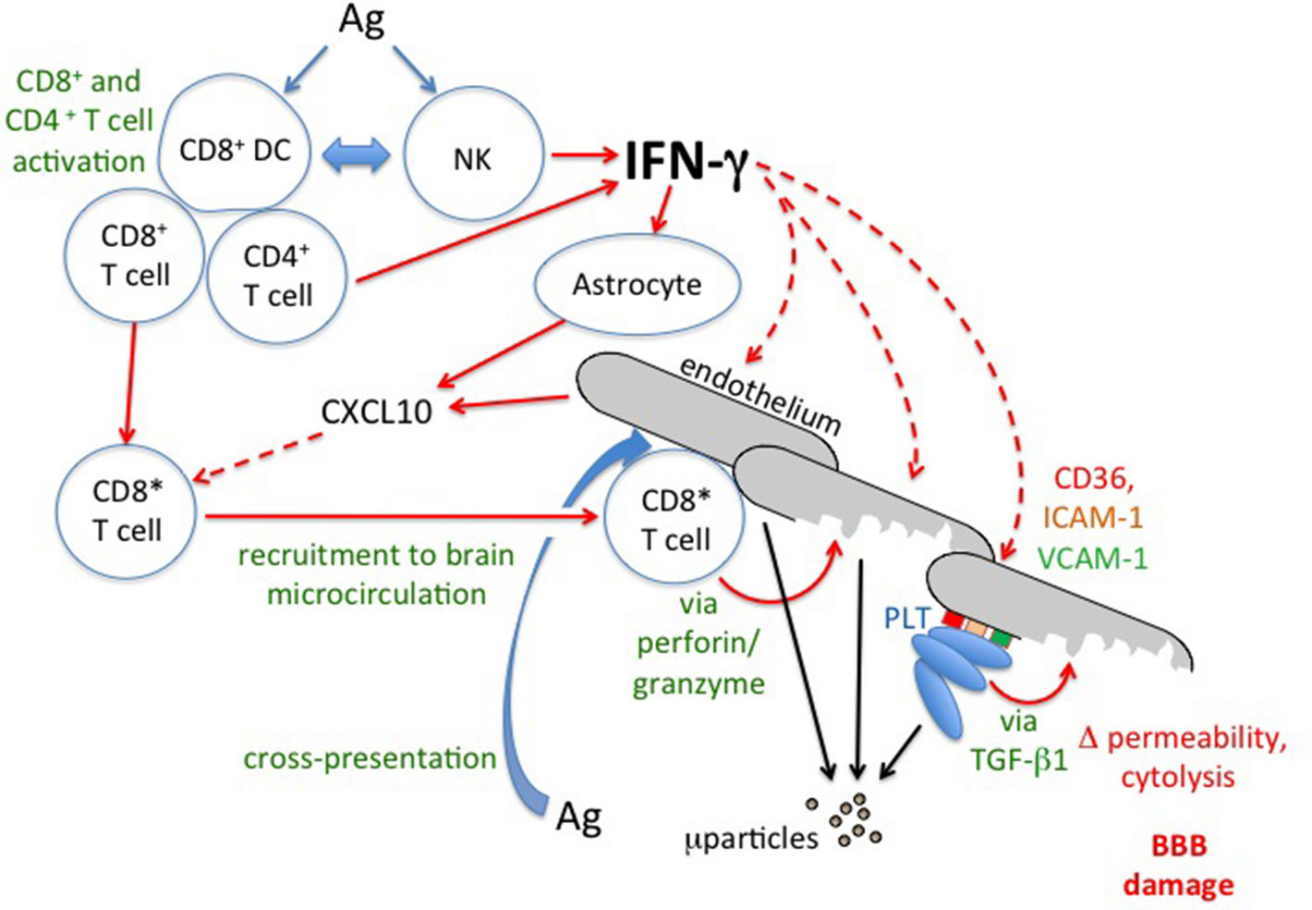
**IFN-γ and the immunopathology of cerebral malaria**. Schematic based on intervention studies in experimental cerebral malaria. For discussion of possible relevance to human cerebral malaria see text. Solid lines indicate direct actions (e.g., release of IFN-γ) or transitions (e.g., activation of CD8^+^T cells to CD8^*^), broken lines indicate influences of secreted factors (IFN-γ and CXCL10). Ag, malaria antigen; BBB, blood-brain barrier; ICAM-I, intercellular adhesion molecule-1; μparticles, microparticles; NK, Natural Killer cell; PLT, platelets; TGF-β1, transforming growth factor-β1; VCAM-1, vascular cell adhesion molecule-1.

Research during the last 25 years has put considerable flesh on the bones of the concept that IFN-γ is a major driving factor in the pathogenesis of cerebral malaria (Figure 9). Immunopathological studies employing interventions, most prominently the use of gene knockout mice, have provided a great deal of molecular information about the multiple levels of IFN-γ involvement in experimental cerebral malaria. There is no similarly comprehensive body of evidence derived from studies of the human condition. However, harking back to undergraduate lectures, “absence of evidence is not the same as evidence of absence.” Some of the relevant correlative evidence reported in clinical or post-mortem studies has been summarized in this article. We contend that further investigation of how the two major proposed mechanisms of cerebral malaria pathogenesis might interact, and the roles of IFN-γ therein, would be beneficial. To this end, we also strongly agree with the sentiment expressed at the 2010 Keystone Symposium on Malaria that “experimental and human studies should be more closely linked so that they inform each other, and that there should be wider access to relevant clinical material” (Langhorne et al., 2011).

### Conflict of interest statement

The authors declare that the research was conducted in the absence of any commercial or financial relationships that could be construed as a potential conflict of interest.
